# 3D-Printed Sensors and Actuators in Cell Culture and Tissue Engineering: Framework and Research Challenges

**DOI:** 10.3390/s20195617

**Published:** 2020-10-01

**Authors:** Pablo Pérez, Juan Alfonso Serrano, Alberto Olmo

**Affiliations:** 1 Instituto de Microelectrónica de Sevilla, IMSE-CNM (CSIC, Universidad de Sevilla), Av. Américo Vespucio, sn, 41092 Sevilla, Spain; pablopg@us.es (P.P.); serrano@imse-cnm.csic.es (J.A.S.); 2 Escuela Técnica Superior de Ingeniería Informática, Departamento de Tecnología Electrónica, Universidad de Sevilla, Av. Reina Mercedes sn, 41012 Sevilla, Spain

**Keywords:** 3D-printed sensors, tissue engineering, electrical impedance spectroscopy, 3D-printed actuators, electrostimulation

## Abstract

Three-dimensional printing technologies have been recently proposed to monitor cell cultures and implement cell bioreactors for different biological applications. In tissue engineering, the control of tissue formation is crucial to form tissue constructs of clinical relevance, and 3D printing technologies can also play an important role for this purpose. In this work, we study 3D-printed sensors that have been recently used in cell culture and tissue engineering applications in biological laboratories, with a special focus on the technique of electrical impedance spectroscopy. Furthermore, we study new 3D-printed actuators used for the stimulation of stem cells cultures, which is of high importance in the process of tissue formation and regenerative medicine. Key challenges and open issues, such as the use of 3D printing techniques in implantable devices for regenerative medicine, are also discussed.

## 1. Introduction

The emergence of rapid prototyping technology and 3D printing makes it possible to quickly develop alternatives to conventional cell culture monitoring infrastructure or tissue engineering equipment which are customizable, low cost, and user-friendly for biological laboratories [1], providing important possibilities in the fields of medical and biological research and clinical diagnostics.

Among the different technologies traditionally used for sensing cell cultures, electrical impedance measurements are one of the most frequently used [2]. Electrical impedance measurements have long been used in biomedical engineering [2] for different applications, such as cell toxicity assessment [3], the study of cell motility [4], analysis of cancer cell lines [5] or tissue engineering [6,7], among others. Electrical cell-impedance sensing, or ECIS, aims at measuring cell impedance, providing information about cell morphology and electric properties, including cell proliferation, attachment, migration, barrier function or cytoplasm conductivity [8]. A common ECIS sensor is composed of a sensing electrode and a reference electrode. Ions move through the cell monolayer between both electrodes, and the measurement of bioimpedance (relation between voltage and measured intensity) is correlated with a specific biological parameter of the cell culture.

Three-dimensional (3D) printing, known as additive manufacturing, describes a family of techniques that involve the fabrication of 3D components using material jetting, powder bed fusion, fused deposition modelling (FDM), sheet lamination, directed energy deposition, photopolymerization, and binder jetting [9]. Some of these three printing techniques are providing the possibility of customization and affordability for biological laboratories for traditional cell culture monitoring applications, as will be reviewed in our work.

On the other hand, three-dimensional (3D) cell culture systems can better reproduce the complex extracellular microenvironments and relations that are produced in tissues or organs and constitute a more precise system to study living organisms in vitro. Bioreactors, manufactured devices that support a biologically active environment, are essential for tissue engineering applications, guiding tissue structure, organization, and functional properties of the biological material through the application of chemical, electrical, or mechanical stimuli. However, conventional imaging techniques are difficult to use in thick samples from 3D cell cultures [10], and new techniques are needed, such as ECIS applications with personalized 3D monitoring setups. In tissue engineering, 3D printing is being increasingly utilized for the design and fabrication of three-dimensional cell culture scaffolds. The integration of sensors and actuators in them is being implemented in order to better control the tissue formation process [10].

In this work, we study the 3D-printed sensors that have recently been used in cell culture and tissue engineering applications, with a special focus on the technique of electrical impedance spectroscopy. Furthermore, we study new 3D-printed actuators used mainly for the stimulation of stem cells cultures, which is of high importance in the process of cell maturation and tissue formation. Finally, new challenges and open issues are discussed, such as the use of new implantable devices for regenerative medicine, based on these same principles and 3D printing.

## 2. 3D-Printed Sensors in Cell Culture and Tissue Engineering

### 2.1. Laboratory Requirements for 3D-Printed Sensors and Bioreactors

The following general requirements are found for 3D-printed sensors and cell culture plates in different laboratory settings:

- Measurement precision: in ECIS measurements, it is necessary to have electrodes with a high conductivity, especially for 2-electrode measurement systems. The counter electrodes must have an adequate sensing area to provide adequate circuit connection [8]. Sensitivity of the electrode must be selected in accordance with the specific biological application to monitor cell culture evolution with the required precision. This precision is also related with the resolution of the printing technology [9].
- Biocompatibility and resistance to biodegradation: both the cell culture substrate and electrode must have biocompatible properties which do not interfere with cell culture evolution [8]. It is necessary to use biocompatible metals, such as Au or silver, for the electrodes, or biocompatible polymers, such as Polylactic Acid (PLA) or Polyethylene Terephthalate Glycol (PETG) [11]. Resistance to biodegradation is also important, taking into account the specific objective and duration of the project [12].
- Cell adherence: 3D-printed plates and electrodes should be treated to provide cell adherence, in a similar way as traditional Petri dishes [8]. For example, FDM has the limitation of the roughness of their surfaces, due to the printing filament width, which could bring difficulties for cell culture growth on its surface [11].
- Microscope visibility: biologists mainly based their observations on the use of microscopy, so an important and desired option is the use of 3D reactors and cell culture-wares that can be easily observed with confocal inverted microscopes. This is translated into transparent surfaces that enable the pass of light through them [11].
- Customization and affordability: for many experiments, it is necessary to have specific cell culture reactors, which can be obtained at a reasonable price for a biological laboratory. There are different companies currently commercializing impedance spectroscopy-based cell culture-wares [13,14], although 3D printing can provide new personalized and affordable solutions to biological labs.

### 2.2. Fabrication Techniques and Materials Used

There are different techniques that can be used for the 3D printing of cell culture electrodes and bioreactors. The most common types are summarized in Table 1. Among the different techniques, the use of multi-material structures with fused deposition modeling provides interesting possibilities, such as the possibility of customization and low costs.

FDM technology makes it possible to quickly develop alternatives to conventional monitoring infrastructure in a customizable, low cost, and user-friendly way. Low resolution and rough surfaces are among the main problems found in this technique, in relation with the need of precise electrodes, cell adherence, and visibility. Among the main materials used in FDM, we can find thermoplastics and conductive thermoplastics, such as PLA, PETG, ABS or PEDOT:PSS. Most of these materials seem to be suitable in terms of biocompatibility, being tested also under in vivo conditions [18]. The use of nanomaterials will improve different properties of the materials used in terms of electrical conductivity, biocompatibility, or even adding new functionalities, such as flexibility or stretchability, which can play an important role in tissue engineering. The integration of nanomaterials in 3D printing technologies is reviewed in [19], for the fabrication of novel electronic, biomedical, and bioelectronic devices. Custom fabricated, biocompatible 3D-printed plastic structures are presented in [20], which can control cell spreading area or medium volume, and exhibit excellent optical properties at 50 µL sample volumes. In [15], a high-performance 3D-printable conducting polymer ink based on PEDOT:PSS, capable of rapid and flexible fabrication of highly conductive microscale structures and devices both in dry and hydrogel states, is presented.

Inkjet-based methods have important advantages, such as high accuracy, high surface finishes and excellent repeatability [21]. However, different disadvantages can be found, such as poor mechanical properties or fragile parts. In 3D inkjet printing, a low viscosity photocurable resin or hydrogel droplets are used as the printing material. Due to its high accuracy, relevant for the manufacturing of high precision sensors and actuators, interesting applications for tissue engineering can be implemented [21].

Powder bed fusion techniques include electron beam melting (EBM), selective heat sintering (SHS), selective laser melting (SLM), and selective laser sintering (SLS). Powder bed fusion methods use either a laser or electron beam to melt and fuse material powder together. These techniques were used to print different bioreactors [22,23], or for the construction of metallic scaffolds in orthopedic implants [24], but they have not been used, to our knowledge, for the fabrication of sensors or actuators in tissue engineering.

In stereolithography, the model is fabricated by curing the 2D layers of a polymer. Resolution of the process can be as precise as 10 µm and, therefore, high-quality precise sensors can be printed [21]. The technique has been widely used for the fabrication of microfluidic devices in diagnostics, but has not been extensively used in tissue engineering. One of the drawbacks is the deleterious effects that UV, used in the curing process, have on cells [25].

### 2.3. Existing Prototypes and Applications

**Figure 1:**
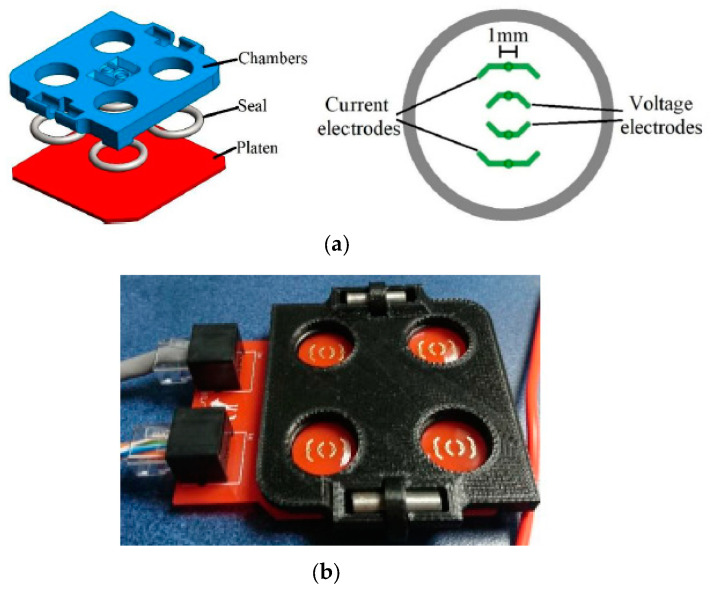
PCB—3D-printed device for the study of impedance spectroscopy in 2D cell cultures. (**a**) Design of the device with four wells for impedance spectroscopy measurements of aqueous solutions and top view of the electrode array of one well. (**b**) Photograph of four 3D-printed reusable wells, each one with 1 mL of an aqueous solution. Reproduced from Cabrera-López et al. [12].

Two-dimensional cell cultures have been traditionally studied with electrical impedance, as previously mentioned, for diverse applications, such as cell toxicity assessment [3], the study of cell motility [4], or tissue engineering [6,7]. In the study of impedance spectroscopy measurements of 2D cell cultures, the use of 3D printing technologies for the implementation of a cell culture device is proposed in Cabrera-López et al. [12]. The proposed device is composed of three pieces (Figure 1a). The support piece of the wells is made on a printed circuit board PCB, on whose top layer an array of gold-coated electrodes was printed (Figure 1b). The middle piece of the framework is composed of four rings that guarantee the hermetic sealing of each well. Finally, the walls of the cell culture wells were manufactured using a FDM 3D-printer. Acrylonitrile butadiene styrene (ABS) material was used to create this chamber, selected because it is not biodegradable (compared with the most commonly used PLA). Initial experiments were carried out with aqueous solutions [12] and erythrocytes [26], with positive results, although electrodes with a lower size were suggested to increase the sensibility to changes of cellular density [26]. A transparent material on the support piece would also be desirable to visually inspect the cell cultures with a confocal inverted microscope.

Joshi et al. [27] presented an electric impedance flow cytometry platform with low-cost inkjet-printed electronics, which can be easily manufactured. The electronics and the microfluidic devices were separate from each other, and a standard inkjet printer was used to print the sensors. The utility of the device to count cells in a sample was demonstrated. Tonello et al. [28] also developed different 2D ink-jet printed sensors (monopolar and interdigitated), and tested them with impedance measurements in fibroblast cell cultures. Sensors were fabricated with a home ink-jet printer, using different biocompatible substrates (Polyimide, PET, PET treated to improve printability, and thermoplastic polyurethane) and conductive inks (PEDOT: PSS, carbon, and silver). PEDOT:PSS and carbon showed better biocompatibility than silver, and polyimide and PEDOT:PSS was selected as the most compatible combination.

**Figure 2:**
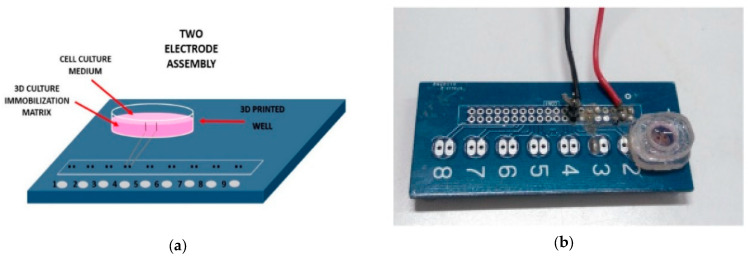
3D-printed PETG well for cell culture monitoring. (**a**) Cross section of the cell chamber with the 3D immobilization matrix. (**b**) Photograph of the well and the electrodes interface. Reproduced from Paivana et al. [29].

Three-dimensional (3D) cell culture systems are being used to better study extracellular environments and reproduce tissue and organ conditions in a more realistic way. Several works have recently used 3D-printed technologies for the study of 3D cultures. In Pan et al. [10], a 3D impedance sensing prototype was developed for the real-time and non-invasive monitoring of 3D cell viability in drug susceptibility studies. Each cell culture well was composed of a pair of vertical gold electrodes, which were laser-cut, bent to a right angle, and attached to the inner surface of the polyethylene terephthalate (PET) culture chamber, fabricated with a numerical control machine. The system showed a good correlation with the conventional imaging methods for monitoring 3D cell viability. In another study [29], two silver (Ag) electrodes were embedded in a 3D-printed PETG well for impedance analysis measurements on immobilized N2a mouse neuroblastoma cells treated with dopamine. Two different cell culture systems were used, allowing either 2D or 3D cell growth (shown in Figure 2). Transparency for optical measurements is described as one of the limitations of the proposed system, and further work is currently being carried out to provide this functionality.

The use of 3D printing to generate customized devices which can support neuronal growth and constrain neurons in defined paths is proposed in [30]. In this article, hardware and software solutions to produce affordable 3D-printed culture devices are reported. Customization is particularly useful for the study of primary neurons, which often require unusual geometries and specialized coatings for optimum growth [30]. As the commercial systems available for culturing primary neurons are expensive and limited in functionality, the proposed technique provides interesting advantages for many areas of neurobiology. The compatibility with live-cell imaging was also shown.

**Figure 3:**
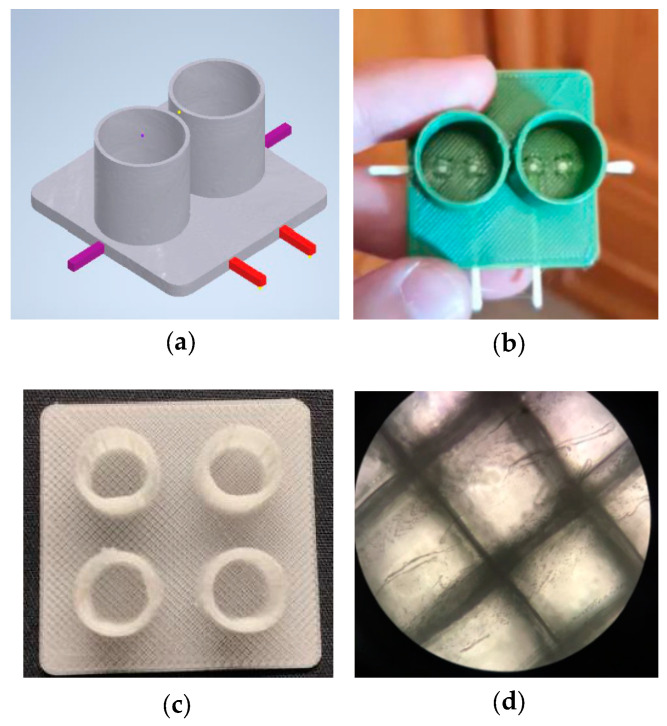
Multi-material 3D-printed bioreactor for cell culture monitoring. (**a**) Bioreactor design. (**b**) Bioreactor implementation in PLA and conductive PLA. (**c**) PETG used as printing material for the bioreactor. (**d**) Microscope image of the PETG surface. Roughness of one of the sides of its surface due to the printing filament is clearly observed. Reproduced from Rodríguez et al. [11].

In [11], an initial design of bioreactor for 2D cell cultures is proposed, with affordable and customized methods. The PRUSA 3D i3 MK3S MMU2S printer was used. The sensors were printed with conductive PLA and integrated in the bioreactor design (Figure 3a,b). The sensing electrode had an area of 1 mm^2^ and a length of 2 cm. Conductivity values for this geometry ranged from 8.8 kΩ to 15.9 kΩ, showing the high impedance measured and the high variability of results obtained. These results suggest that other geometries should be designed for electrodes, with larger contact areas and shorter lengths. This would lead to reduced electrical resistance, but also reduced electrical resistance variability. The microscope visibility of different materials (PLA, PETG) was also tested, PETG being a good candidate for this (Figure 3c,d). Although visibility was good enough for microscopes, the rough surface found due to the printing filament can raise several problems for cell adherence; a solution was found with the printing of different pieces and using flat surfaces. Further work is needed to test the 3D-printed bioreactor with cell cultures and assess the validity of the proposed bioreactor for cell culture monitoring.

The combined use of electronics and tissue engineering constructs is presented in Mannoor et al. [31]. In this work, a 3D bionic ear was printed with cartilage tissue, and an electrically conductive silver nanoparticle inductive coil antenna was attached to the printed ear to catch electromagnetic signals. Xu et al. [32] fabricated 3D elastic membranes shaped to match the epicardium of the heart, as a deformable platform for arrays of multifunctional sensors, in an interesting example in the field of cardiology.

## 3. 3D-Printed Actuators in Tissue Engineering

In tissue engineering, the control of tissue formation is very important to form tissue constructs that can be used in clinical practice [33]. Optimization of tissue engineering to develop functional muscle requires a complex strategy combining metabolic optimization, stimulation with soluble factors, and biophysical stimulation [34].

Electrical pulse stimulation (EPS) has been applied to induce cell clustering in cultured neural networks [35] or has improved skeletal muscle regeneration through satellite cell fusion with myofibers in healthy elderly subjects [36], among other applications. In [33], electrical pulse stimulation (EPS) was used to improve skeletal muscle development and maturation. In this work, it was shown how important the geometry of electrodes or the type of signal used in electrostimulation can be, producing different cellular development and tissue formation. Other types of cell stimulation include heat stimulation [37] or mechanical stimulation [38]. Ultrasonic stimulation has also been used to improve stem cell proliferation and differentiation [39].

The emergence of 3D printing technologies makes it possible to quickly develop alternatives to conventional stimulation infrastructure which are low cost, customizable, and simple to use. Three-dimensional printing provides important possibilities in the specific field of electrostimulation, enabling the development of personalized bioreactors, which include 3D electrostimulators that can adapt to the specific application targeted in tissue engineering. Three-dimensional multi-material printing technologies can provide important personalization features for the implementation of 3D actuators in tissue engineering, using 3D scaffolds used in the process of creation of tissue also as a good architecture for the colocation of printed actuators. Initial work in 3D-printed devices for electrostimulation can be found in [30]. In this work, it is described how to use printed 3D devices to grow and stimulate neurons. PLA was chosen versus Acrylonitrile Butadiene Styrene (ABS) for economic and simplicity reasons. This work provides different examples to illustrate the practical utility and potential that these protocols can bring for many aspects of experimental neurobiology. In [1], a rapid prototype of a three-dimensional (3D)-printed reusable growth chamber with integrated electrodes for electrical stimulation is also described. The model can easily be reconstructed within a few hours using 3D desktop printing and off-the-shelf components and was initially tested with the cell line MED17.11 as a model system.

**Figure 4:**
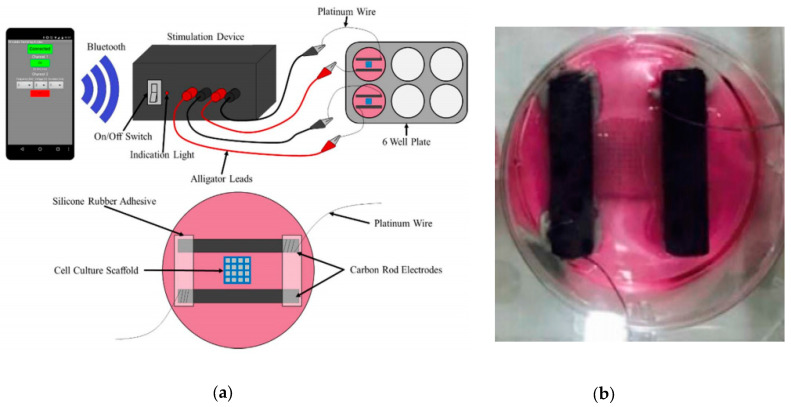
3D-printed electrostimulator for cardiomyocyte cell cultures. (**a**) Complete setup for electrical stimulation and detail of a single well. (**b**) Photograph of the experimental setup used in one well, with the cell culture scaffold in the middle of the carbon electrodes. Reproduced from Scott et al. [40].

Initial prototypes are also proposed in the cardiovascular research field. In [40], a technique is presented for the rapid design and fabrication of scaffolds from silicone rubber and polycaprolactone (PCL) for primary human cardiomyocyte cell cultures. Additionally, a stimulation device is developed, providing wirelessly controlled electrical stimulation (5 V, 2 ms pulses, 1 Hz) to the cell culture scaffolds (Figure 4). The results indicate the effectiveness of both the scaffold fabrication technique and the stimulation device, showing higher cell attachment and differentiated actin cytoskeletal structures compared to the unstimulated scaffold. Although further study is needed to optimize the setup for a better control of the different biological parameters, the potential of this technique in tissue engineering applications is presented.

In [41], the use of 3D-printed graphene/PCL scaffolds for both in vitro and in vivo bone remodeling applications was investigated. The scaffolds were used to treat rat calvaria defects using micro electrical stimulation (10 μA). The proposed scaffolds and electrostimulator increased cell migration and inflammatory cell influx, achieving a better tissue formation and bone remodeling.

Agarwala et al. [37] proposed a novel 3D bioprinting approach to fabricate bioelectronic platforms, based on flexible gelatin methacrylate and silver nanoparticle ink electrodes. These electrodes were employed for electric and heat stimulation, and also for sensing cell culture assays. Putame et al. [38] applied 3D printing (FDM) for the in-house production of customized components of a mechanical stretching bioreactor. Potential application for cardiac tissue engineering and mechanobiology studies were described. Future manufacturing in biocompatible and autoclavable materials is envisioned.

Finally, low-intensity ultrasound can also enhance the proliferation and differentiation of stem cells during the process of 3D bioprinting [39]. In this work, the use of ultrasound to improve three-dimensional (3D) bioprinting for the construction of complex and heterogeneous tissue structures is presented. Acoustic impedance and physical properties of 3D-printed ultrasound sensors are also interesting and emerging topics [42].

Table 2 summarizes the most relevant applications found in these sections for 3D printing in cell culture monitoring and tissue engineering stimulation applications, with a summary of the materials used, its main uses, main advantages, and reported limitations.

As can be seen in these examples and applications, 3D printing techniques have recently shown important possibilities in the field of cell culture and tissue engineering, with good potential to be used to improve tissue constructs and functional behavior of biological material. Affordability and customization are the most important advantages found. Affordability is especially important in 2D and 3D cell culture monitoring, to reduce costs in biological laboratories. Customization is the main advantage found in electrostimulation applications, where 3D printing technologies have initially proved their advantages over existing laboratory instrumentation. There is still room for improvements that can achieve a more personalized and complete control of tissue engineering processes.

## 4. Key Challenges and Open Issues

We have analyzed the different requirements found for 2D cell culture monitoring with impedance spectroscopy, including finding the need for biocompatible materials, cell adherence, visibility to microscopes, customization, or affordability. FDM has emerged as a promising technology to implement affordable and personalized sensing bioreactors. Low resolution and rough surfaces are among the main problems and challenges found in FDM, although new improvements in multi-material printers will help overcome these limitations. Stereolithography and powder bed fusion techniques have also been used to print high-quality bioreactors and microfluidic devices for diagnostics. Inkjet printing, on the other hand, has recently been proposed for high-precision sensing electrodes, also with low-cost instrumentation. Improving poor mechanical properties and fragile parts and integrating these inkjet-printed sensors in 3D-printed bioreactors are two of the remaining challenges.

In the field of 3D culture and tissue engineering, different works have been reviewed, showing the benefits of personalization and affordability brought by 3D printing. In all articles reviewed in the electrostimulation field, results obtained with the 3D-printed prototypes showed improvements over the traditional ones (cell proliferation, tissue formation, etc.). Furthermore, 3D-printed actuators are also presented for other types of simulation, such as mechanical, by means of heat, or with ultrasonic waves. Undoubtedly, many other works will appear during the next years, showing the possibilities of these technologies in research and in future clinical use in tissue engineering. A more careful study on biocompatible materials is, in many cases, needed, as well as autoclavable materials for commercial uses.

Another important field is the fabrication of implantable devices for regenerative medicine, a further step that can be carried out using the same principles studied here. In [43], an implantable electrical stimulator for improving osteoblast proliferation and differentiation is presented, consisting of a flexible electrode and a triboelectric nanogenerator. Initial experiments have been carried out in rats, demonstrating that this electrical stimulator improves cell adherence, cell proliferation, differentiation processes, and the regulation of calcium ions. In [44], a new design of biocompatible silicone enclosures for implantable medical microdevices is presented, studying its use in deep brain stimulation microdevices and demonstrating its potential for neurosurgery applications. Additionally, in this field, in [45], a two-dimensional array of polyethylene glycol (PEG)-tipped microneedles is presented for confining microparticles for brain and soft tissue implantation. In vivo experiments, where microparticles were implanted into a rat cortex, were described, discussing several issues, such as high-throughput implantation challenges, tissue injury caused by the method, or spatial stability of implanted devices under chronic conditions. Minimizing tissue injury is proposed by treating microneedles with dexamethasone [45].

In [46], a technique for fabricating cardiac microphysiological devices with multi-material (3D) printing is presented. Specifically, six functional inks were fabricated based on high-conductance and biocompatible soft materials. Targeting also cardiovascular applications, different implanted devices for the monitoring of the stent occlusion status were proposed in [47], and the potential use of 3D printing technologies for the fabrication of these devices was studied in [48], highlighting specific materials, such as PEDOT:PSS or flexible printed electrodes. However, further work is still required to test biocompatibility and functional behavior of these designed devices under in vivo situations. Other important engineering problems found in the practical application of implanted intelligent stents, such as requirements of accuracy, reliability, low power, and reduced size, were described in [49] and will have to be addressed in each specific medical or clinical application. Artificial intelligence algorithms for real-time monitoring 3D-printed electrical impedance tomography (EIT) sensors have been recently presented, applied to the monitoring of lung deformation [50]. The use of data analytics also constitutes an emerging topic in the field of real-time sensors, and will play an important role in tissue engineering and implantable devices.

## 5. Conclusions

Three-dimensional printing makes it possible to quickly design and implement alternatives to conventional ECIS electrodes and cell culture bioreactors that are customizable, low cost, and user-friendly. Among the main requirements for 3D-printed bioreactors to be employed in cell culture monitorization, we can find the need for biocompatible materials, cell adherence, visibility to microscopy, customization, or affordability. FDM has emerged as a promising technique for the implementation of personalized and affordable sensing bioreactors. Low resolution and rough surfaces are among the main problems found, although a number of successful devices for different applications are described. Inkjet printing has also recently been proposed for manufacturing high-precision sensing electrodes, also with low-cost instrumentation. New advances in printing nanomaterials will provide improved properties, such as better conductivity, biocompatibility, or even new functionalities, such as flexibility or stretchability.

Three-dimensional printing also provides important possibilities in the specific field of regenerative medicine and electrostimulation, enabling development of personalized bioreactors which include 3D stimulators that can adapt to a specific objective. Interesting applications are described in the fields of neurobiology, cardiac tissue engineering, and bone remodeling. The different works studied showed very positive results for the use of this technology in biotechnological laboratories, although there is still room for new improvements that can achieve a more personalized and complete control of tissue engineering processes.

Finally, the different works analyzed also propose the use of 3D printing technologies for implantable sensors and actuators in different fields of regenerative medicine (osteoblast regeneration, cardiac tissue formation and monitorization devices, or deep brain stimulation). Potential use of the technology is presented, although further work is required to test biocompatibility and functional behavior under in vivo situations. Three-dimensional technology improvements will also allow us to make a reality these envisioned applications for implantable devices in the next few years.

## Figures and Tables

**Table 1:** Main 3D printing technologies, advantages and limitations.

3D Printing Technology	Materials Used	Advantages	Limitations	Main Uses	Ref.
Fused deposition modeling (FDM)	(Conductive) thermoplastics (PLA, PETG, PEDOT:PSS, ABS, etc.)	Multi-material (heterogeneous) structures, biocompatibility, low cost.	Low resolution, rough surface (low cell visibility and adherence)	Cell chamber, bioreactor container	[11] [12]
Inkjet based methods (material jetting, binder jetting)	Liquid photopolymers	High accuracy, excellent repeatability, heterogenous materials	Poor mechanical properties, fragile parts	Electrode fabrication	[15]
Powder bed fusion (SLM, SLS, SHS, EBM, etc.)	Powdered plastic, metal, ceramic, etc.	High accuracy, high strength, biocompatibility,	High cost, limited mechanical properties	Metallic scaffolds, bioreactor container	[16]
Stereolithography	Photopolymers	High accuracy, heterogenous materials	Low biocompatibility	Electrochemical sensors	[17] [13]

**Table 2:** Main applications found, advantages and limitations.

Application	Use and Materials	Advantages	Reported Limitations	Ref.
2D cell culture monitoring	Cell chamber (PLA, PETG, ABS), sensing electrode (conductive PLA)	Affordability, customization	Precision, rough surface, non-visibility to microscope	[12] [11]
3D cell culture monitoring	Cell chamber (PLA, PET) sensing electrode (PCB)	Affordability, customization	Non-visibility to microscope	[10] [29] [30]
Neuron electrostimulation	Cell chamber (PLA)	Affordability, customization	Possible long-term toxicity	[30] [1]
Cardiomyocyte electrostimulation	Cell chamber (silicone rubber/PCL) Stimulation electrode (carbon rod)	Customization, improved cell proliferation	Poorer cell adherence in silicone, more detailed analysis needed	[40]
Bone electrostimulation	Scaffolds fabrication and electrostimulation electrode (graphene/PCL)	Customization, improved cell proliferation	---	[41]
Electrical and heat stimulation	Cell chamber (gelatin methacrylate hydrogel) Stimulation electrodes (nanoparticle silver ink)	Affordability, customization	Low proliferation index in primary human fibroblasts	[37]
Mechanical stimulation of cells/tissues	Cell culture chamber housing (ABS)	Customization, facility to assemble and use	Low biocompatibility, unautoclavable materials	[38]
